# Age-related changes of fibroblast density in the human periodontal ligament

**DOI:** 10.1186/1746-160X-9-22

**Published:** 2013-08-21

**Authors:** Elena Krieger, Sandra Hornikel, Heinrich Wehrbein

**Affiliations:** 1 Department of Orthodontics, Medical Centre of the Johannes-Gutenberg-University Mainz, Augustusplatz 2, Mainz 55131, Germany

**Keywords:** Fibroblast, Periodontal ligament, Aging, Human, Orthodontic

## Abstract

**Objective:**

Recently, research has focused intensely on age-related tissue changes, not only in the field of dermatology but also in dental sciences. Although many new insights into age-related morphological, ultrastructural and biochemical changes in the periodontal ligament tissue have been gained, the basic question of whether there is a quantitative change in cell number remains unanswered or, at least to date, unpublished. Thus, the aim of this study was to detect age-related changes of the periodontal ligament regarding fibroblast density.

**Material and methods:**

33 lateral tooth-bearing segments of the maxilla were obtained from deceased human individuals of different age, ranging from 7 to 63 years. The buccal segment of the periodontal ligament of the mesiobuccal root of the first maxillary molar was evaluated histomorphometrically to obtain the fibroblast density.

**Results:**

The results clearly indicate a steady and statistically significant decline of fibroblast number with age.

**Conclusion:**

It may be concluded that fibroblast density in the physiological human periodontal ligament tissue decreases with age, thus causing an initial delay in physiological, pathological or externally induced processes that require remodeling of the periodontal ligament, e.g. traumatic occlusion or orthodontic tooth movement. It may be assumed that an orthodontic tooth movement in elderly patients requires more time in the initial treatment phase and should be done with lighter forces.

## Introduction

Recently, research has focused intensely on age-related tissue changes, not only in the field of aesthetic dermatology but also in the dental field. Judging from clinical experience, relevant changes were suspected in the periodontal tissue thus influencing orthodontic tooth movement and the progression of periodontal disease.

Evidence was found that orthodontic tooth movement seems to be impaired in adult patients, thus requiring different treatment protocols than in adolescent patients. These findings will be discussed more thoroughly later in this article.

Periodontal disease however seems to be less dependent on age [1]. A study comparing experimental gingivitis in young vs. old individuals by Fransson et al. [2,3] did confirm that older individuals experience more severe inflammation in response to experimental gingivitis. Nevertheless, several studies evaluating whether age is a risk factor for increased loss of periodontal support, have shown that aging either has no effect at all or, if so, the effect is minor and clinically insignificant [4-8] and that smoking has much more influence than age [9].

Research efforts are focused today on age-related biochemical, morphological, ultrastructural and genetic changes at the cellular or subcellular level of the periodontal tissue. Current in-vitro models for evaluating age-related changes take different approaches. Some authors employ primary cell cultures from donors of different ages while others take cells from higher passages of the same cell line. Zeng et al. [10] even take the approach of working with young versus old cell culture medium thus focusing on the extracellular environment as a major influencing factor for age-induced changes. In-vivo models usually comprise of studying young versus aged animals with subsequent sacrificing and histological evaluation - the currently preferred animal model being the rat.

Many authors have found evidence that aged periodontal ligament cells show a higher basal level of inflammatory-related genes [11,12] as well as an increased expression of inflammatory mediators following exposure to mechanical stress [13-15].

Nishimura et al. [16] found that the replicative capacity of cells from aged donors was impaired. This however does not lead to an accumulation of senescent cell populations in the periodontal ligament of aged donors as Sawa et al. [17] showed. Moxham and Evans [18] even found ultrastructural changes in periodontal ligament cells from aged animals indicating a decrease in collagen producing activity.

Some descriptive studies on cell numbers were published at the beginning of the age-related research era. Grant and Bernick [19] evaluated specimens obtained from four human male cadavers (age 55-92y) and found an age-related increase in fibrosis and a decrease in cellularity of the periodontal ligament tissue. Severson et al. [20] evaluated 24 human cadavers, ranging in age from 20 to 90y, in a descriptive manner. In the older specimens they found a decrease in fiber and cellular content in the periodontal ligament tissue.

The decrease in cellular elements was observed by Levy et al. [21] also in an animal study evaluating the periodontal ligament of young versus old marmoset monkeys. Berglundh at al. [22] evaluated the periodontal ligament tissue of 10 beagle dogs in two different age groups (1y vs. 8-9y) and found that in the older dogs the fibroblasts occupied less volume (13%) than in the younger dogs (22%).

Nevertheless, in the light of all the new insights gained about age-related changes in the human periodontal ligament itself, the question of whether there is a quantitative change in cell density remains unanswered or, at least to date, unpublished.

## Material and methods

In this study tooth-bearing lateral segments of the maxilla were obtained from 33 deceased human individuals for evaluation. The individuals were between 7 and 63 years of age, 22 male and 11 female. Inclusion criteria were the following: no crowns or bridges, full antagonist dentition, tissue sampling and fixation within 12 h post mortem. The specimens were obtained from the Institute of pathology and the Institute of forensic medicine at the University of Aachen, Germany, after the required authorization was given by the legally responsible person.

All 33 individuals were categorized into age segments. The definition of segments, the number of individuals per age segment as well as the average age in each segment are listed in Table 1.

**Table 1:** Definition of age segments and number of individuals per age segment

**Age segment**	**No. 1**	**No. 2**	**No. 3**	**No. 4**	**No. 5**	**No. 6**
Age range (in years)	0-9y	10-19y	20-29y	30-39y	40-49y	> = 50y
Number of individuals	1	4	8	11	6	3
Average age in segment (in years)	7.0	17.0	23.6	33.7	45.1	54.7

**Figure 1:**
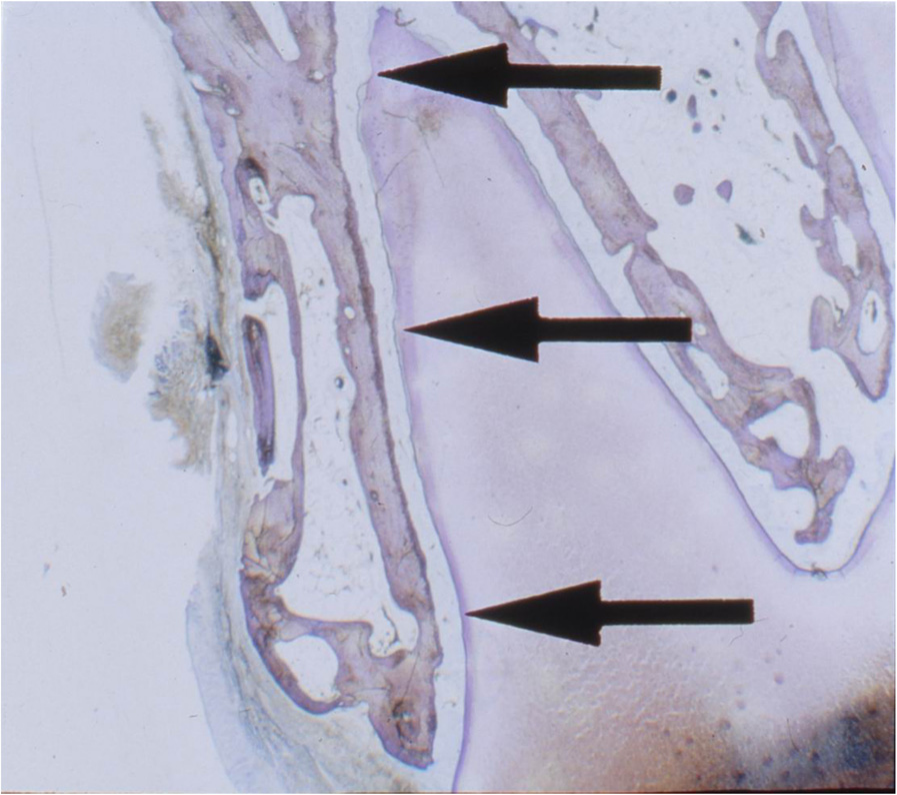
**Histological sample of the buccal periodontal ligament of the mesiobuccal root of the first upper molar.** Original magnification 2.5×.

All segments of the maxillae were cut transversally into 1–2 mm thick slices in the region of the first upper molar. Then the tissue was kept in a fixative (neutral buffered formalin) for 48 h. Subsequently the specimens were processed using the cutting-grinding technique until 5–15 μ thick sections were obtained. Finally, the specimens were stained with toluidine blue (Figure 1).

### Histomorphometry

The specimens were evaluated by histomorphometry which, by definition, is a quantitative study of the microscopic organization and structure of tissue (such as bone) particularly by means of computer-assisted analysis of images formed by a microscope.

In our study the histomorphometry was carried out with a computer (IBM, Armonk, USA), a SummaSketch digitizer and a digitizing tablet (Summagraphics Corporation, Lansdale, Pennsylvania USA) along with the appropriate software and a microscope (Leica Microsystems, Wetzlar, Germany). The magnification used was 390.6×.

For this analysis we evaluated the specimen showing the buccal part of the periodontal ligament of the mesiobuccal root of the first upper molar. The measurements were carried out in three height segments of the periodontal ligament: the apical third, the middle third and the incisal third. The apical measurement was taken approx. 2 mm to the incisal of the apex; the incisal measurement was taken approx. 1–2 mm to the apical of the alveolar bone ridge while the middle measurement was taken midway between the first two measurements.

**Figure 2:**
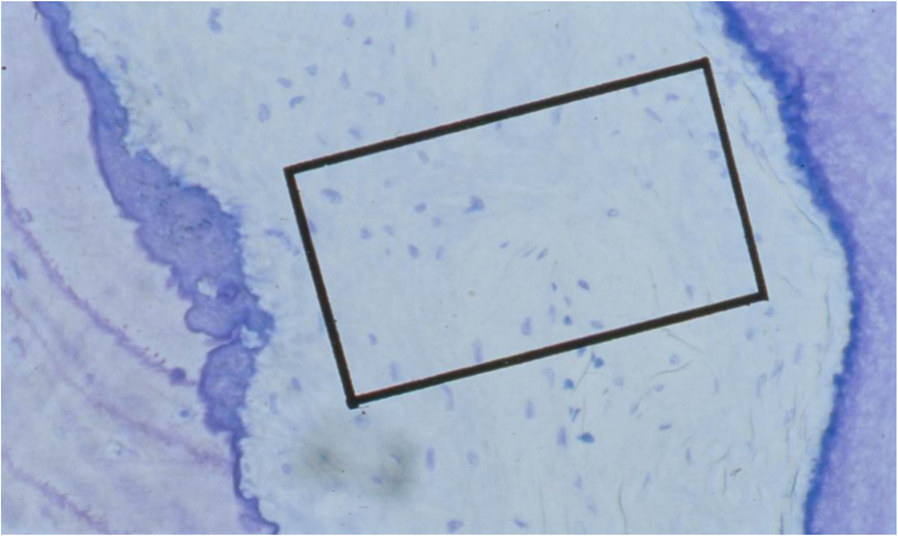
**Optical cell counting grid projected onto histological specimen stained with toluidine blue.** Original magnification 390.6×.

An optical grid of 2×5 squares (18 766 μm^2^) was projected onto the periodontal ligament at the appropriate height ensuring that the grid neither overlapped the alveolar bone nor the root cementum (Figure 2). The number of fibroblasts within the grid was counted. After this, the grid was moved slightly and counting repeated twice at each height. Using these data sets, the computer calculated an average number of fibroblast cells for each area.

### Statistics

Statistics were computed with the SAS software (SAS Institute, Cary, USA). For the age-related changes in the number of fibroblasts we computed an average of the measurements taken at three levels of the periodontal ligament for each individual. In order to detect incisal-apical differences in the periodontal ligament we analyzed a height-specific comparison over all age segments. For statistical significance the Wilcoxon test was performed with p < = 0.05 rated as significant.

## Results

The fibroblast density i.e. the average number of fibroblast cells in a defined area (18766 μm^2^) declines steadily with age. In a 7-year-old individual it is 51.5. Up to the 6th age segment it declines by more than half to 19.9. This change is statistically significant between the 2nd and 3rd as well as between the 3rd and 6th segments (p < =0.05) (Table 2).

**Table 2:** Average fibroblast density per age segment

**Age segment**	**Average number of fibroblast cells**	**Standard deviation**	**Minimum**	**Maximum**
No.1	51.1	-	-	-
No.2	40.4	13.4	25.5	57.3
No.3	29.9	6.5	16.6	41.3
No.4	28.2	4.9	20.2	37.2
No.5	24.6	3.4	17.5	28.0
No.6	19.9	6.6	14.0	27.0

## Discussion

Oehmke et al. [23] investigated physiological age-dependent changes in the periodontal ligament of molars in rats (1, 8 and 18 months of age) regarding collagen fiber formation. They found an age-related decrease in the amount of collagenous fiber production, but could not clearly attribute it to a decreased number of fibroblasts or a decreased collagen production rate per cell. The results of our study suggest that a reduced number of fibroblasts is one of the factors which induce a reduced amount of collagenous fibers. Regardless of age, the authors found that the formation of collagen was enhanced in the apical and cervical thirds compared to the middle third of the periodontal ligament. They regarded this fact as a response to functional strain.

Kyomen and Tanne [24] investigated the age-related changes of the proliferation rate of periodontal ligament cells during experimental tooth movement in rats. Experimental animals were divided into two groups: young rats (6 weeks old) and adult rats (14 weeks old). A control group was allotted to each group. Light forces (10 g) and heavy forces (40 g) were applied to the first upper molar over a time period of 1 to 14 days. The authors found a significant difference in proliferation rate between young and adult animals in the early stages of tooth movement. But they also found a corresponding difference of proliferative activity in young and adult control animals. They concluded that these findings suggest a delayed biological response to orthodontic tooth movement in the initial phase, which is age-related.

Ren et al. [25] studied orthodontic tooth movement over a wider range of ages, the youngest rats being 6 weeks old and the adult rats being 9–12 months old, as well as over a longer period of time. The study was conducted using a split-mouth design and applying a standardized force of 10 cN to 3 molars over 1 – 12 weeks. The authors found that tooth movement in adult rats was only slower in the initial phase. Later on both age groups underwent a similar amount of tooth movement. The authors published another aspect of this study in 2008 [26]. They found that the surface area of the periodontal ligament was smaller on the pressure side than on the tension side at week 1 in young rats, while it was smaller at week 8 in adult rats. They concluded that disorganization and reorganization of the periodontal ligament tissue occurred earlier in young rats than in the adults. The results of this study may also be explained in part by the decreased number of fibroblasts initially available in adults, which corresponds to the results of our study.

It may be concluded from the results of our study that the fibroblast density in the physiological human periodontal ligament tissue decreases with age, thus causing an initial delay in physiological or induced processes that require remodeling of the periodontal ligament, e.g. traumatic occlusion, orthodontic tooth movement etc.

## Conclusion

It may be concluded from the results of our study that fibroblast density in the physiological human periodontal ligament tissue decreases with age, thus causing an initial delay in physiological, pathological or externally induced processes that require remodeling of the periodontal ligament, e.g. traumatic occlusion, orthodontic tooth movement etc. Regarding a clinical relevance, it may be assumed that an orthodontic tooth movement in elderly patients requires more time in the initial phase of the treatment and should be done with lighter forces than in young patients. Also, in periodontal concerns a reduced fibroblast density should be expected in elderly patients.

## Competing interests

The authors declare that they have no competing interests.

## Authors’ contributions

EK, SH, HW conceived the study design, assembled the data, conducted the analysis and interpretation of data, and drafted the manuscript. All authors read and approved the final manuscript.
